# Influence of Upconversion Processes in the Optically-Induced Inhomogeneous Thermal Behavior of Erbium-Doped Lanthanum Oxysulfide Powders

**DOI:** 10.3390/ma9050353

**Published:** 2016-05-11

**Authors:** Rolindes Balda, Noha Hakmeh, Macarena Barredo-Zuriarrain, Odile Merdrignac-Conanec, Sara García-Revilla, M. Angeles Arriandiaga, Joaquín Fernández

**Affiliations:** 1 Departamento de Física Aplicada I, Escuela Superior de Ingeniería, Universidad del País Vasco UPV-EHU, Alda. Urquijo s/n, 48013 Bilbao, Spain; macarena.barredo@ehu.eus (M.B.-Z.); sara.garcia@ehu.eus (S.G.-R.); joaquin.fernandez@ehu.eus (J.F.); 2 Materials Physics Center CSIC-UPV/EHU and Donostia International Physics Center, 20080 San Sebastián, Spain; 3 Laboratoire des Verres et Cerámiques, UMR-CNRS 6226, Université de Rennes, Campus de Beaulieu, 35042 Rennes Cedex, France; Noha.Hakmeh@univ-rennes1.fr (N.H.); odile.merdrignac@univ-rennes1.fr (O.M.-C.); 4 Departamento de Física Aplicada II, Facultad de Ciencia y Tecnología, Universidad del País Vasco UPV/EHU, Apartado 644 Bilbao, Spain; mariaangeles.arriandiaga@ehu.eus

**Keywords:** laser materials, upconversion, low phonon materials, laser cooling, erbium

## Abstract

The efficient infrared-to-visible upconversion emission present in Er-doped lanthanum oxysulfide crystal powders is used as a fine thermal sensor to determine the influence of upconversion processes on the laser-induced thermal load produced by the pump laser and to assess the potentialities of this material in order to obtain anti-Stokes laser-induced cooling. The analysis of the upconversion emission and excitation spectra as well as the decay curves indicates that energy transfer upconversion is the main mechanism responsible for the green (^4^S_3/2_) and red (^4^F_9/2_) upconversion luminescence. The dependence on temperature of the intensity ratio of upconversion emission from thermally-coupled ^2^H_11/2_ and ^4^S_3/2_ levels of Er^3+^ in the 240–300 K temperature range has been used to estimate a relative sensitivity of 1.09 × 10^−2^ K^−1^. Thermal measurements performed on the powder samples by using a thermal infrared camera exhibit a very inhomogeneous heat distribution at the sample surface due to the random distribution of the pumping energy inside the sample as well as to the random properties of the thermal field. The analysis of both spectroscopic and thermal measurements show that after a transient heating induced by the background absorption, cooling of discrete regions by means of anti-Stokes processes can be observed.

## 1. Introduction

The first evidence of anti-Stokes laser-induced cooling in erbium-doped solid samples, a KPb_2_Cl_5_ crystal and a fluorochloride glass, were provided by some of the authors in 2006 [1]. It is worthy to mention that cooling was observed in the spectral region where some upconversion processes starting at the pumped level occur [1,2]. Moreover, in a more recent work [3], we have also reported experimental evidence of the fact that the efficient infrared-to-visible upconversion present in Nd^3+^-doped KPb_2_Cl_5_ crystal and powder can lead to local internal and bulk optical cooling. These results show that new cooling channels based on upconversion processes can be of paramount importance in the search of new rare-earth-doped materials for optical cooling.

There are two main reasons that hinder the cooling efficiency in rare-earth (RE) doped solid: the nonradiative transitions between the energy levels of the RE ion and the presence of impurities in the host matrix that gives rise to parasitic absorptions generating heat. To overcome these limitations, host materials with low phonon energies have been used. As a rule of thumb, a negligible impurity parasitic absorption and near-unity quantum efficiency of the anti-Stokes emission from the RE levels involved in the cooling process are required.

The investigation of new hosts for rare earth ions with low phonon energies is still an open problem with very important implications from both the fundamental and practical points of view. Though fluoride compounds have been extensively studied due to their low phonon energy, chloride- and sulfide-based hosts present the advantage of a lower phonon energy that leads to a significant reduction of the multiphonon relaxation rates. However, these materials usually present poor mechanical properties and moisture sensitivity, and it is difficult to grow large-sized and very pure single crystals. This difficulty could be overcome by exploring the possibility of obtaining laser cooling in a powder made up from nano- to micro-sized rare earth-doped crystals. Laser-induced cooling has been already demonstrated by some of the authors in a Nd-doped KPb_2_Cl_5_ crystal powder [3], in agreement with the predictions of a previous theoretical model [2]. The results showed that the inhomogeneous nature of these systems introduces new dependencies of the cooling experiment on the grain sizes and their spatial distribution. In particular, the light diffusion of the pumping light inside the sample, induced by multiple scattering processes, may produce localized-like energy regions inside the sample and as a consequence subsequent cooling and/or heating discrete zones.

In this work we analyze the optical and thermal response of Er^3+^-doped La_2_O_2_S crystal powders by exciting the ^4^I_9/2_ level of Er^3+^ ions. The low phonon maximum energy (~400 cm^−1^) lanthanum oxysulfide crystal is a wide-gap semiconductor material known as an excellent host for trivalent rare-earth ions [4,5,6]. A detailed study of the upconversion processes and temperature field exhibited by the Er^3+^-doped powder sample by pumping the ^4^I_9/2_ level is presented. The analysis of both spectroscopic and thermal measurements show that after a transient heating induced by the background absorption, cooling of discrete regions by means of anti-Stokes processes can be observed.

## 2. Experimental

### 2.1. Samples

The synthesis procedure of the Er-doped oxysulfide powders was the following: the starting materials La(NO_3_)_3_·6H_2_O (Alfa Aesar, 99.9%, GmbH Co. KG, Karlsruhe, Germany), Er(NO_3_)_3_·H_2_O (Alfa Aesar, 99.99%, GmbH Co. KG, Karlsruhe, Germany), and thioacetamide CH_3_CSNH_2_ (Aldrich ≥ 99.9%, Sigma Aldrich, Saint-Quentin-Fallavier, France) were dissolved in absolute ethanol (Prolabo, Normapur, 20 mL, VWR, Strasbourg, France). Stoichiometric lanthanum nitrate/fuel molar ratio was used in all preparations. The solution was heated below 80 °C to allow the dissolution of thioacetamide. The preparation was then introduced into a muffle furnace (Thermolyne 48,000) pre-heated to 500 °C. After the inflammation reaction of ethanol, a spontaneous combustion reaction characterized by a high flame temperature, produced an expanded white solid. This product was finally ground and post-treated under a H_2_S/N_2_ flow at 1000 °C for two hours. Fourier transform infrared (FTIR) analysis has been performed on the powders before and after H_2_S/N_2_ treatment in order to assess the purity of the powders. FTIR transmittance spectra of all samples present several absorption peaks between 400 and 4000 cm^−1^. As reported in a previous study of La_2_O_2_S:Er^3+^,Yb^3+^ [7], the absorption peaks are assigned to water, carbonate, and sulfate groups. After sulfurization, all these peaks disappear, except La−O and La−S vibrational bands situated at 400–550 cm^−1^. La_2_O_2_S powders containing 0.5, 1, 2, 3, and 6 mol % of Er^3+^ were prepared with a filling factor of 0.15.

### 2.2. Experimental Techniques

The steady-state emission and excitation spectra were recorded by exciting the samples with a continuous wave (cw) Ti:sapphire ring laser (0.4 cm^−1^ linewidth) in the 770–920 nm spectral range. The fluorescence was analyzed with a 0.25 m monochromator, and the signal was detected by a Hamamatsu R636 photomultiplier and finally amplified by a standard lock-in technique. Infrared (IR) emissions were detected with an extended IR Hamamatsu H10330A-75 photomultiplier. The sample temperature was varied between 240 and 300 K with a continuous flow cryostat.

Lifetime measurements were obtained by exciting the samples with a dye laser pumped by a pulsed nitrogen laser and a Ti-sapphire laser pumped by a pulsed frequency-doubled Nd:YAG laser (9 ns pulse width), and detecting the emission with Hamamatsu R636 and H10330A-75 photomultipliers. Data were processed by a Tektronix oscilloscope.

## 3. Near-Infrared Emission

**Figure 1:**
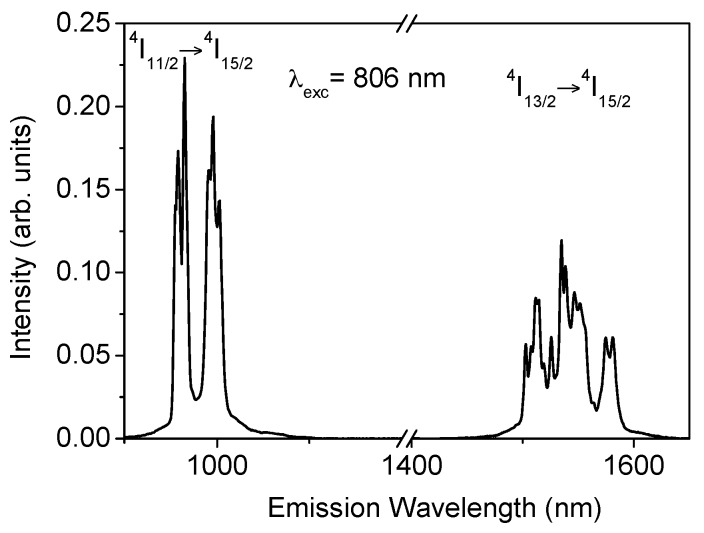
Room temperature emission spectrum obtained under excitation at 806 nm (*λ*_exc_ is the excitation wavelength).

The near-infrared (NIR) emission was obtained for all samples at room temperature by exciting at 806 nm in the ^4^I_9/2_ level. After excitation of this level, the next lower levels are populated by radiative and nonradiative relaxation. As an example, Figure 1 shows the fluorescence spectrum corresponding to the ^4^I_11/2_ → ^4^I_15/2_ and ^4^I_13/2_ → ^4^I_15/2_ transitions for the sample doped with 2 mol% of Er^3+^. As can be seen, the ^4^I_11/2_ → ^4^I_15/2_ emission shows an intensity higher than the ^4^I_13/2_ → ^4^I_15/2_ transition. The energy separation between ^4^I_11/2_ and ^4^I_13/2_ levels is around 3500 cm^−1^ which corresponds to nine phonons and, therefore, the multiphonon relaxation rate between levels ^4^I_11/2_ and ^4^I_13/2_ is very low.

**Figure 2:**
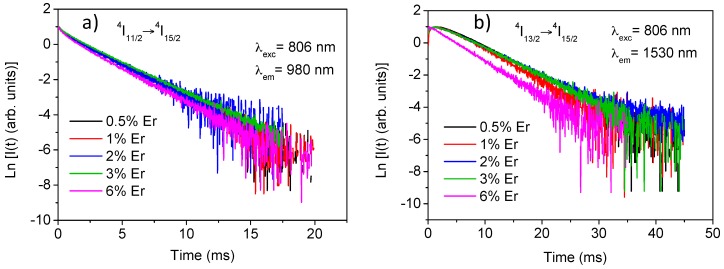
Semilogarithmic plot of the experimental decays of the ^4^I_11/2_ (**a**) and the ^4^I_13/2_ (**b**) levels for all samples obtained under excitation at 806 nm.

The experimental decays of luminescence from levels ^4^I_9/2_, ^4^I_11/2_, and ^4^I_13/2_ were obtained at room temperature for all samples by exciting at 806 nm in level ^4^I_9/2_ and collecting the luminescence at 822, 1000, and 1530 nm respectively. The lifetime values for the three levels are displayed in Table 1. Due to the energy gap between levels ^4^I_9/2_ and ^4^I_11/2_ (around 2000 cm^−1^), the lifetime of the former is strongly reduced by multiphonon relaxation processes giving a value of around 13 μs. The decays of the ^4^I_11/2_ level slightly deviate from a single exponential behaviour and the lifetime is nearly independent on concentration up to 3 mol% and decreases for the highest concentrated sample. The lifetime values correspond to the fitting to a single exponential function. In the case of the ^4^I_13/2_ level, the decay shows an initial rise time due to the population from the higher ^4^I_11/2_ level and a single exponential behavior at all concentrations. The lifetime value remains constant for Er^3+^ concentrations between 0.5 and 3 mol% and decreases to 4.6 ms for the highest concentrated sample. Concentration quenching strongly depends on the quality of the materials achieved by the synthesis process. In this system the high purity and well-crystallized samples minimize the quenching processes. Figure 2a,b show the experimental decays for ^4^I_11/2_ and ^4^I_13/2_ levels for all concentrations.

## 4. Near-Infrared to Visible Upconversion in Er^3+^-Doped La_2_O_2_S Crystal Powders

**Figure 3:**
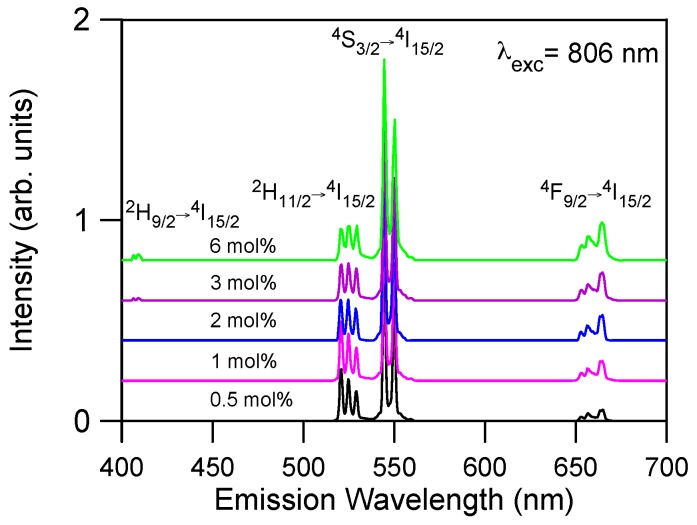
Room temperature upconversion emission spectra under excitation at 806 nm for all concentrations. Spectra have been shifted vertically to ease comparison.

In this section we present an analysis of the upconversion mechanisms in Er^3+^-doped La_2_O_2_S crystal powders following excitation into the ^4^I_9/2_ level by using both steady-state and time-resolved laser spectroscopy. The upconverted emission spectra obtained under cw excitation were measured by using a Ti-sapphire ring laser. Cut-off filters were used to remove the pump radiation. Figure 3 shows the upconverted emission spectra of Er^3+^ doped La_2_O_2_S obtained under excitation at 806 nm in resonance with the ^4^I_15/2_ → ^4^I_9/2_ transition for all concentrations. The observed emissions correspond to transitions ^2^H_11/2_ → ^4^I_15/2_, ^4^S_3/2_ → ^4^I_15/2_, and ^4^F_9/2_ → ^4^I_15/2_. The ^2^H_11/2_ → ^4^I_15/2_ emission can be observed at room temperature because the ^2^H_11/2_ level is populated from ^4^S_3/2_ via fast thermal equilibrium between levels. As can be observed, the most intense emission corresponds to the green emission from level ^4^S_3/2_. The samples doped with 3 and 6 mol % show a weak blue emission around 410 nm corresponding to the ^2^H_9/2_ → ^4^I_15/2_ transition. The weak red emission from the ^4^F_9/2_ level also increases as Er^3+^ concentration increases. This emission can not be attributed to multiphonon relaxation from level ^4^S_3/2_ since the energy gap between both levels is about 2800 cm^−1^ and the maximum phonon energy is around 400 cm^−1^. The strongest green upconversion emission corresponds to the highest concentrated sample.

To investigate the excitation mechanisms for populating the ^4^S_3/2_ and ^4^F_9/2_ levels after NIR excitation, we have obtained the evolution of the upconverted emission intensities. Upconversion intensities were recorded at 546 and 665 nm for different pump powers. The upconversion emission intensity (I_em_) depends on the incident pump power (P_pump_) according to the relation I_em_ ∝ (P_pump_)^n^, where n is the number of photons involved in the pumping mechanism. The dependence of the intensity on the pump power is nearly quadratic which indicates a two photon (TP) upconversion process to populate the ^4^S_3/2_ and ^4^F_9/2_ levels.

As it is well known, the most important upconversion processes are ground state absorption (GSA) followed by excited state absorption (ESA) and energy transfer upconversion (ETU). The way to distinguish between ESA and ETU processes is the excitation spectra (under cw excitation) and the time evolution of the upconversion luminescence after pulsed excitation. In the case of GSA/ESA a single ion is involved, whereas two ions are involved in an ETU process. In this last case, the excitation spectrum is similar to the GSA absorption spectrum of the intermediate state, whereas in the case of excited state absorption the upconversion excitation spectrum is the result of GSA absorption and excited state absorption and may differ from the GSA of the intermediate state.

**Figure 4:**
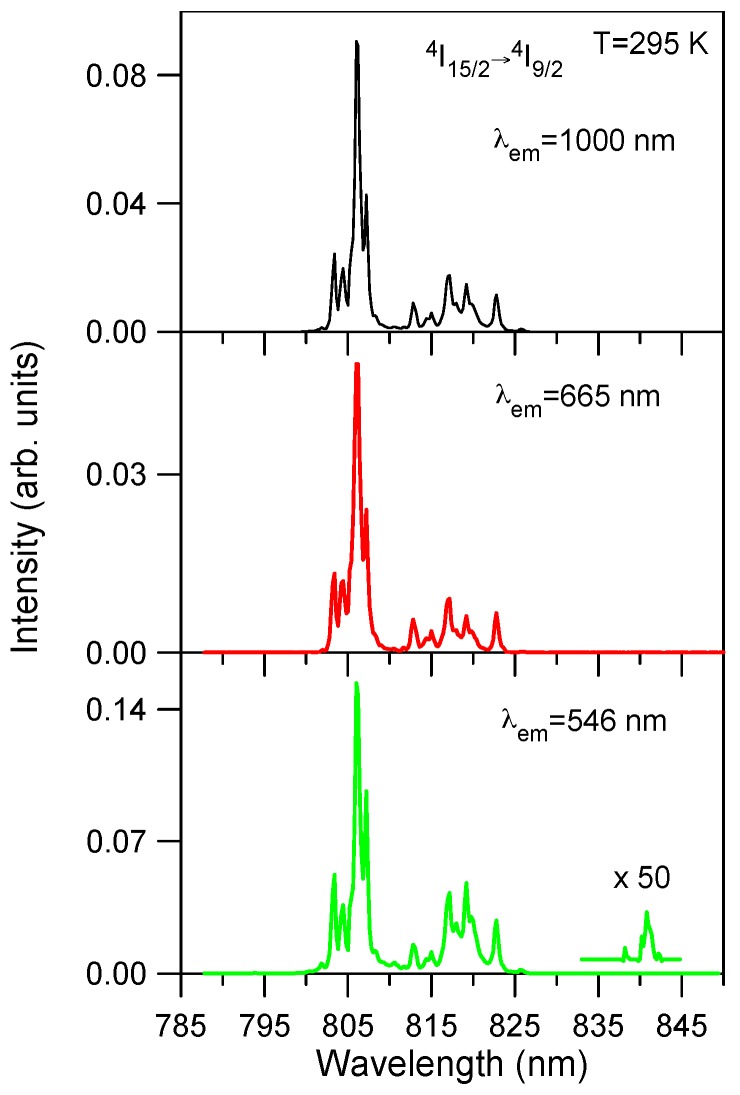
Excitation spectra of the upconverted emissions from levels ^4^S_3/2_ (546 nm) and ^4^F_9/2_ (665 nm) corrected for the spectral variation of the laser intensity. The ground state excitation spectrum corresponding to ^4^I_11/2_ → ^4^I_15/2_ transition is also included for comparison.

Excitation spectra of the green emission at 546 nm (^4^S_3/2_) and the red emission at 665 nm (^4^F_9/2_) have been performed in the spectral region corresponding to the ^4^I_15/2_ → ^4^I_9/2_ transition. Figure 4 shows the excitation spectra of the green and red emissions for the lowest concentrated sample (0.5 mol %) together with the ground state excitation spectrum of the infrared emission from level ^4^I_11/2_ for comparison. As can be seen the upconverted excitation spectra follows the same wavelength dependence as the ground state excitation spectrum. The same behavior is observed for all concentrations. This fact suggests that energy transfer processes are dominant for the upconverted luminescence even for the low concentrated samples. The upconversion excitation spectrum of the green emission shows one additional weak peak at around 842 nm which does not appear either in the infrared spectrum or in the one photon ground state absorption spectrum. This feature can indicate the presence of ESA processes since GSA and ESA steps occur at slightly different wavelengths. The energy difference between the maximum of the spectrum and this weak peak corresponds to the energy carried out by one phonon (~400 cm^−1^) of the host matrix.

The time dependent behavior of the upconverted luminescence under pulsed excitation depends on the upconversion process. The radiative ESA process leads to an immediate decay of the upconversion luminescence with the same lifetime than that obtained after direct excitation, whereas in the case of ETU the decay curve for the upconversion emission, shows a rise time after the laser pulse and a lifetime longer than the one after direct excitation. This distinction is possible when the pulse width is much shorter than the time constant of the relevant energy transfer step [8]. The rise time is influenced by both the energy transfer processes and the lifetime of the emitting state. The decay reflects the slow feeding of the high excited state by energy transfer between two excited Er^3+^ ions.

**Figure 5:**
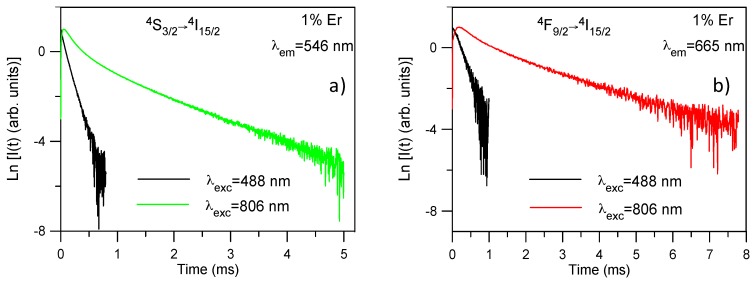
Semilogarithmic plot of the experimental decays of the (**a**) green emission from the ^4^S_3/2_ level and (**b**) red emission from the ^4^F_9/2_ level obtained under excitation at 488 nm and 806 nm.

To confirm if ETU processes are dominant to populate the ^4^S_3/2_ and ^4^F_9/2_ levels, we have obtained the decay curves of the green and red emissions under pulsed (9 ns pulse width) infrared excitation at 806 nm when the emitting states are populated via the ^4^I_11/2_ state. The decays and lifetimes of the ^4^S_3/2_ and ^4^F_9/2_ levels have also been obtained under direct excitation with a dye laser. For all samples, the decays obtained under NIR excitation show longer lifetimes than those observed under direct excitation which suggests again the presence of ETU processes. As an example, Figure 5a shows the time evolution of the upconverted ^4^S_3/2_ emission at room temperature under 488 and 806 nm excitation for the sample doped with 1 mol%. The decay after 488 nm excitation can be described by a single exponential function; however, after 806 nm excitation the decay shows a short rise time (*τ*_rise_) with a two-exponential decay behavior. The lifetimes of the two components of the decay (*τ_s_* and *τ_l_*) are much longer than that of level ^4^S_3/2_ under direct excitation which is a fingerprint of ETU. A similar behavior is observed for all concentrations (Table 2). As can be seen in Table 2, the lifetimes for both excitation wavelengths decrease as Er^3+^ concentration increases probably due to the presence of cross-relaxation processes such as ^2^H_11/2_ → ^4^I_9/2_ and ^4^I_15/2_ → ^4^I_13/2_ transitions [9].

**Figure 6:**
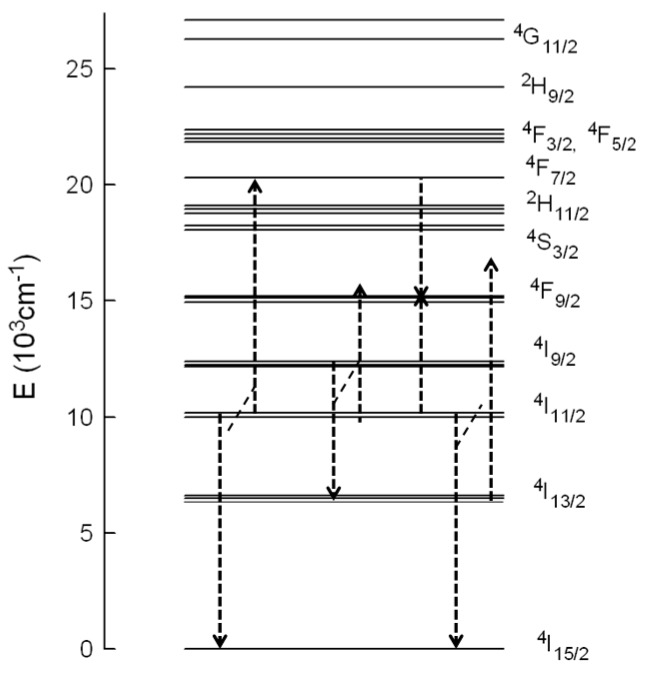
Energy level diagram and possible ETU mechanisms to populate ^4^S_3/2_ and ^4^F_9/2_ levels.

The lengthening of the upconverted green emission lifetime compared to the lifetime after direct excitation indicates the presence of ETU processes since the decay is influenced by the lifetime of the emitting level and the lifetime of the levels feeding the ^4^S_3/2_ state through the energy transfer process. According to the energy level diagram of Er^3+^ in this material (Figure 6), the ^4^S_3/2_ level can be populated by ESA and/or ETU processes. The absorption of one NIR pump-photon excites the electrons to level ^4^I_9/2_; then, considering the short lifetime of this level, multiphonon relaxation occurs to level ^4^I_11/2_ and subsequent ESA of a second NIR pump photon promotes the electrons to the ^4^F_3/2,5/2_ levels until, finally, by nonradiative relaxation, ^2^H_11/2_ and ^4^S_3/2_ levels are reached. Part of the excitation energy in the ^4^I_11/2_ level further relaxes to level ^4^I_13/2_. Under this excitation condition, ESA from level ^4^I_13/2_ to ^2^H_11/2_ can occur. ETU processes such as (^4^I_11/2_ → ^4^I_15/2_) and (^4^I_11/2_ → ^4^F_7/2_) can also be responsible for the green emission. In this process two Er^3+^ ions in the ^4^I_11/2_ level interact, and one ion gains energy and reaches the ^4^F_7/2_ level, whereas the other one loses energy and goes to the ground state. In this case the lifetime of the decay should be about half the lifetime of the ^4^I_11/2_ level. Since the lifetime is shorter than half the lifetime of the intermediate level, we can not disregard the presence of ESA processes. Considering that, in the case of ESA, the lifetime of the ^4^S_3/2_ level should be the same as the one measured after direct excitation, it is possible to estimate the ESA and ETU contribution to the total intensity [10]. If the area under the decay with the intrinsic lifetime of the green emission under 488 nm excitation represents the ESA contribution, the obtained values for this contribution in the upconversion emission decays change from 21.5% (0.5 mol %) to 11% (6 mol %).

The decays of the ^4^F_9/2_ → ^4^I_15/2_ transition obtained under NIR excitation also show a rise time, longer than in the case of the green emission, followed by a two-exponential decay. The values for rise and decay times are shown in Table 3. As an example, Figure 5b shows the decays under 488 nm and 806 nm excitations for the red emission from level ^4^F_9/2_ for the sample doped with 1 mol %. The upconversion decay curve is markedly different from the decay obtained under one photon excitation. The lifetime of the long component is about 1.5 ms which is much longer than the one of the level under direct excitation which confirms that ETU processes are dominant to populate the ^4^F_9/2_ level. The rise of the upconversion decay after the excitation pulse is another clear indication of ETU. According to the energy level diagram of Er^3+^ in this matrix, there are different ETU processes to populate this level (Figure 6). In the first case, one ion in the ^4^I_9/2_ state decays to the ^4^I_13/2_ state and transfers its energy to another ion in the ^4^I_11/2_ state which reaches the ^4^F_9/2_ level (energy mismatch +843 cm^−1^). In the second process, two Er^3+^ ions interact, one of them in the ^4^I_11/2_ level and the other one in the ^4^F_7/2_ level, both going to level ^4^F_9/2_. This process has an energy mismatch of around +260 cm^−1^. There exists another possible mechanism to populate the ^4^F_9/2_ level involving these transitions; in this process one ion in the ^4^I_11/2_ level goes to the ground state and transfers its energy to the other one in level ^4^I_13/2_, which reaches level ^4^F_9/2_; however, this mechanism has the highest energy mismatch (+1657 cm^−1^).

As can be seen in Table 3, the rise time drops one order of magnitude for the highest concentrated sample. This behavior could be related with the higher initial population of the ^4^I_11/2_ intermediate state at this concentration. The higher population reduces Er^3+^-Er^3+^ distances and increases energy transfer processes giving rise to a fast upconversion.

## 5. Upconversion Luminescence Thermometry by Using Emissions from Thermally-Coupled Er^3+^ Levels

The fluorescence intensity ratio (FIR) method utilizes pairs of levels with a small energy difference in which the high energy level is thermally populated from the lower energy one. As we have mentioned before, the green emission corresponds to transitions from the two thermally-coupled ^2^H_11/2_ and ^4^S_3/2_ levels. The energy difference between ^2^H_11/2_ and ^4^S_3/2_ levels in the present host is about 715 cm^−1^, which fulfills the requirements for the applications of the FIR method [11]. The fluorescence intensity ratio resulting from the emissions of these two thermally-coupled levels can be derived from the Boltzmann distribution by the following equation [11]:

(1) FIR=I(H11/22)I(S3/24)=gHAHνHgSASνSexp(−ΔEKT)=Bexp(−ΔEKT)

where *g_H_* and *g_S_* are the degeneracies of the ^2^H_11/2_ and ^4^S_3/2_ levels respectively, *A_H_*, *A_S_*, *ν_H_*, and *ν_S_* are the spontaneous emission rates and frequencies of the ^2^H_11/2_ → ^4^I_15/2_ and ^4^S_3/2_ → ^4^I_15/2_ transitions, respectively. This equation can be expressed as follows:

(2) $$Ln\left(FIR\right)=Ln\left(B\right)-\frac{\Delta E}{KT}=Ln\left(B\right)-\frac{C}{T}$$

where *B* and *C* are constants to be determined.

For sensing applications it is important to know the rate at which the fluorescence intensity ratio changes for a small temperature change. This quantity known as the sensitivity *S* is defined as:

(3) $$S=\frac{d\left(FIR\right)}{dT}=FIR\frac{\Delta E}{KT^{2}}$$

This equation shows that, at a given temperature, a larger energy gap between two thermally-coupled energy levels gives a larger *S*, and a smaller temperature variation can be detected by measuring the change of *FIR*. The temperature resolution *R* indicates the minimal detectable signal change and can be estimated from the standard deviation of residuals in the fit of the experimental points and the calculated sensitivity *S* [12]:

(4) $$R=\frac{\Delta \left(FIR\right)}{S}$$

**Figure 7:**
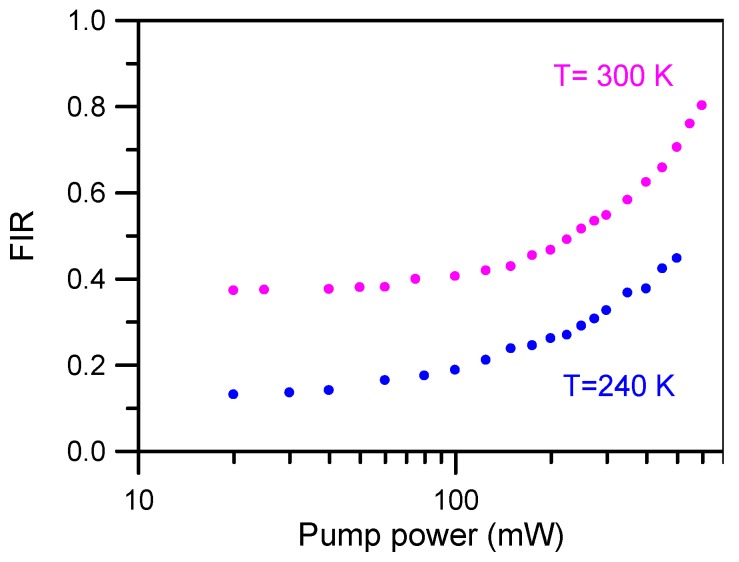
Fluorescence intensity ratio as a function of pumping power at two different temperatures.

In order to calibrate the fluorescence intensity ratio of these emissions *versus* temperature, the green upconversion emission spectra of the sample doped with 2 mol % of Er^3+^ were performed from 300 K downwards to 240 K by pumping at 806 nm. To avoid the heating effect produced by laser excitation, the upconverted green emission has been measured at different pump powers at two different sample temperature (240 K and 300 K). As can be seen in Figure 7, at low excitation power there is no effect on the spectra, but for pumping powers higher than 50 mW the effect is not negligible. Considering these results, an unfocused laser beam of 40 mW power at 806 nm was selected to obtain the fluorescence intensity ratio.

**Figure 8:**
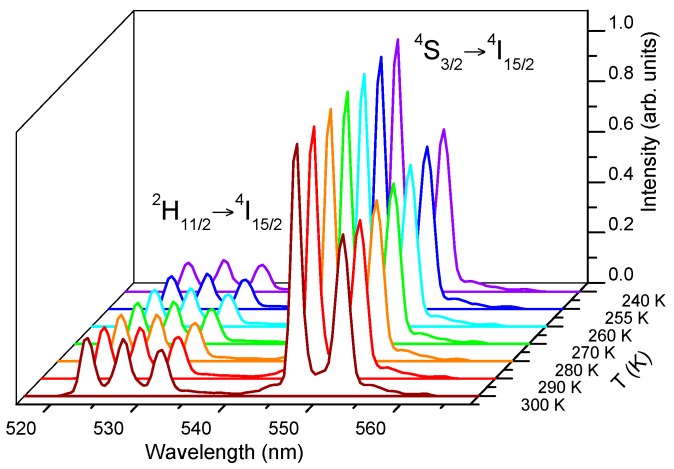
Upconverted green emission as a function of temperature in the 300 K–240 K temperature range obtained under excitation at 806 nm.

**Figure 9:**
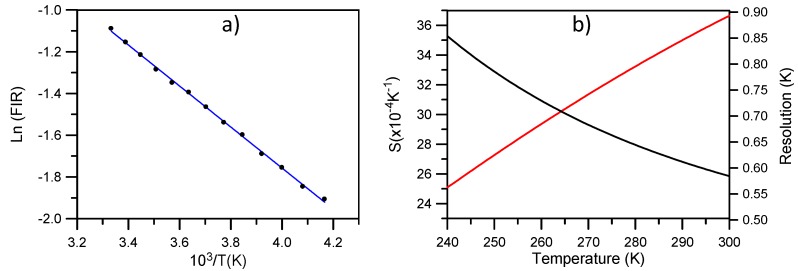
(**a**) Integrated intensity ratio of the green upconverted emissions as a function of 10^3^/T in the 300 K–240 K temperature range. The solid line corresponds to the fitting with Equation (2); (**b**) Sensitivity (red line) and resolution (black line) of the FIR temperature sensor based on La_2_O_2_S:Er^3+^.

Figure 8 shows the green upconversion emission at different temperatures between 240 K and 300 K obtained under excitation at 806 nm keeping the pump power constant at 40 mW. As the temperature increases, the emission of the ^2^H_11/2_ level gradually increases with respect to the ^4^S_3/2_ level due to the increase of the population of the ^2^H_11/2_ level at the expense of ^4^S_3/2_, according to the exponential dependence with temperature given in Equation (1). As can be seen in Figure 9a, the fitting of the integrated fluorescence intensity ratio of transitions from the ^2^H_11/2_ and ^4^S_3/2_ levels to the ground state with Equation (2) shows a good correlation between experiment and theory. The obtained parameters are *Ln(B)* = 2.17 and *C* = 983. The experimental value of *ΔE/K* is 1017 in good agreement with the fitting parameter.

Figure 9b shows the absolute sensitivity *S* (in K^−1^) of the measurement together with the maximal temperature resolution achievable in theory. As we can see, for temperatures higher than 255 K we have a resolution better than 0.6 K. The relative sensitivity defined as *S_R_ = ΔE/KT*^2^ gives a value of 1.09 × 10^−2^ K^−1^ at room temperature similar to the one obtained for NaYF_4_ [13].

To further investigate the relationship between excitation wavelength and sample temperature we have performed emission spectra by exciting at different wavelengths in the low (842 nm) and high (790 nm) energy sides of the excitation spectra. These wavelengths have been chosen in order to keep the same pump power absorption taking account for the absorbance profile of the transition. The excitation at 842 nm corresponds to the weak peak observed in the excitation spectrum of the green upconverted luminescence. The results show that the emission from level ^2^H_11/2_ slightly increases with respect to the ^4^S_3/2_ level by exciting at 790 nm at the high energy side of the ^4^I_15/2_ → ^4^I_9/2_ absorption band. By using the obtained relationship between the integrated fluorescence intensity ratio of transitions ^2^H_11/2_ → ^4^I_15/2_ and ^4^S_3/2_ → ^4^I_15/2_ and temperature, the sample temperature when exciting at 790 nm is 4.1 K higher than under excitation at 842 nm. As will be confirmed in the next section, it is possible to obtain laser cooling under anti-Stokes excitation at 842 nm aided by upconversion processes.

## 6. Thermal Study of Er^3+^ Ions in La_2_O_2_S Crystal Powders

To assess the potential cooling capabilities of Er^3+^ ions in lanthanum oxysulfides powders we have used an infrared thermal camera (FLIR SC7500-MB) working in the 2.5–5.1 μm spectral range with a sensitivity of around 20 mK. The crystal powder was compacted in a 6 mm high cylindrical quartz cell (6 mm diameter) without a front window for handling ease and optical characterization. The volume filling factor of the powder material (*f* = 0.15) was calculated by measuring the sample volume and weight. The average grain size distribution of the microstructured powder is about 3 μm although the grains are composed of nanometer sized (~100 nm) particulates. The sample was placed in low vacuum inside a cryostat chamber provided with a wide NIR transparent window through which the camera lens allows focusing on the sample surface. The thermal scans were recorded at a rate of 5 Hz. The absolute sample temperature was calibrated with a thermocouple.

**Figure 10:**
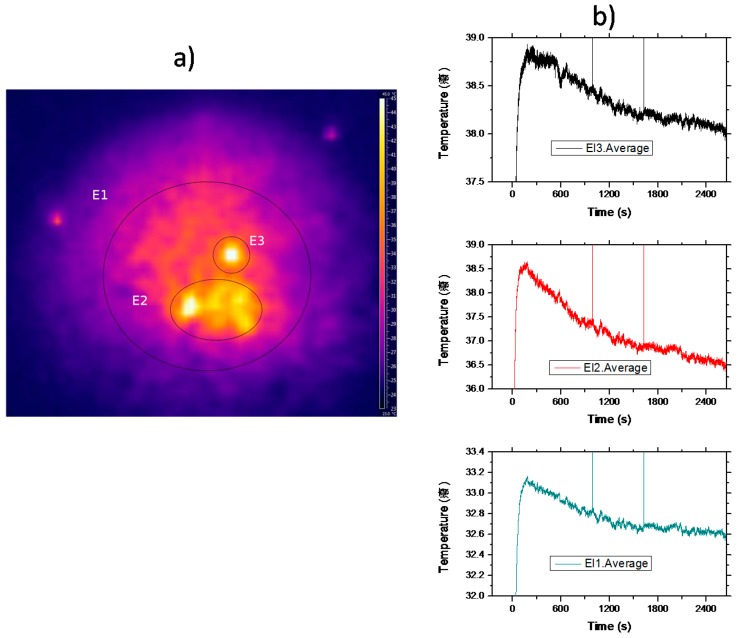
(**a**) FLIR camera video frame showing discrete thermal zones after pumping with 300 mW at 842 nm in a 2 mol% Er^3+^- doped La_2_O_2_S powder sample; (**b**) Average temperature as a function of time measured in the three shown zones in (**b**).

As we have seen above, the spectroscopic results obtained by pumping the sample in the anti-Stokes region at 842 nm, with an energy mismatch of about 400 cm^−1^ (one phonon energy) below the barycenter of the absorption band, suggest that the sample may cool when pumped in this region. Following this hint, we have analyzed the thermal response of the sample by pumping with a tunable femtosecond laser working at 80 MHz (1 W pump power at 842 nm). Figure 10a shows a typical video frame of the sample after irradiation. As can be seen, the temperature distribution is rather inhomogeneous showing a sharp distribution of hot spots. Although the excitation beam profile is Gaussian, due to the inhomogeneous nature of crystal powders, the light diffusion of the pumping light inside the sample, induced by multiple scattering processes, may produce localized-like energy regions inside the sample and, as a consequence, subsequent cooling and/or heating discrete zones. The thermal pattern remains quite the same all along the experiment. Figure 10b displays the average temperature as a function of time measured in the three shown zones. Initially, the temperature of the sample rises in all the zones from room temperature (~24 °C ) until it reaches a stationary regime when the thermal load deposited on the material is compensated by the fluorescent losses. As can be seen, in the hot zone, E2, the temperature falls by 2 °C in forty minutes, whereas in the wider one, E1, the average temperature drops about 0.5 °C. These results are in good agreement with the expected random propagation of radiation in a region with a randomly distributed dielectric constant. Moreover, the static disorder produces well defined propagation modes inside the sample, in agreement with the discrete static heating observed at the sample surface. Finally, it is worthy to point out that the average temperature of the sample may cool after the initial transient heating, induced by the background absorption, due to the infrared-to-visible upconversion processes that can offset the heat load deposited in the doped powder.

**Figure 11:**
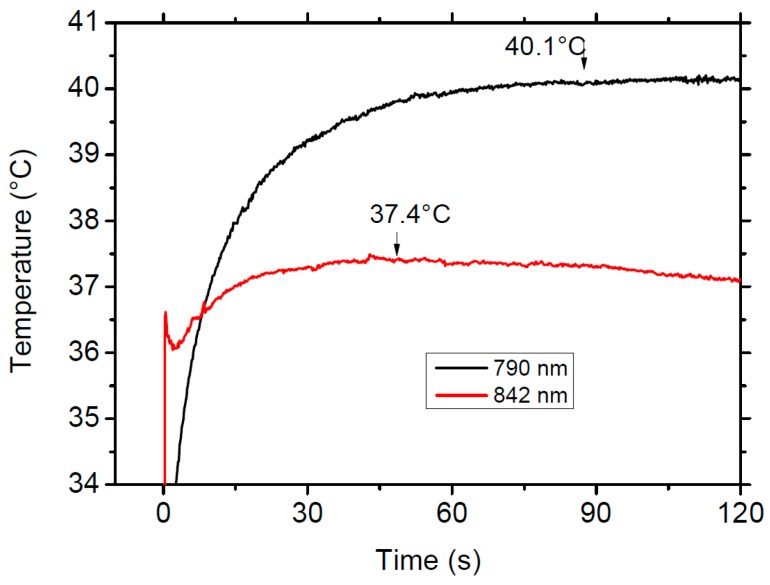
Comparison of the thermal response of the sample when excited at 790 nm, in the Stokes side (black line) of the absorption spectrum and in the cooling region, at 842 nm (red line), under the same experimental conditions with a power of 700 mW.

As we have mentioned in the previous section, by using the obtained relationship between the integrated fluorescence intensity ratio of transitions ^2^H_11/2_ → ^4^I_15/2_ and ^4^S_3/2_ → ^4^I_15/2_ and temperature, the sample temperature when exciting at 790 nm is 4.1 K higher than under excitation at 842 nm. A similar temperature difference is observed in the thermal response of the sample under excitation in the Stokes (790 nm) and anti-Stokes (842 nm) side of the absorption band. Figure 11 shows the comparison of the thermal response of the sample when excited at 790 nm, in the Stokes side (black line) of the absorption spectrum, and in the cooling region, at 842 nm (red line), under the same experimental conditions with a pump power of 700 mW. The figure clearly shows the different thermal behavior of the sample in the heating and cooling regions.

## 7. Conclusions

A detailed spectroscopic study of the excitation and emission spectra and time-resolved upconversion luminescence of Er^3+^ ions in lanthanum oxysulfide powders shows that ETU processes are dominant in the population of the ^4^S_2/2_ and ^4^F_9/2_ levels under near infrared excitation in the ^4^I_9/2_ level even for the lowest concentrated sample (0.5 mol %). We have experimentally demonstrated that the efficient infrared-to-visible upconversion processes can be used as a fine temperature thermometer to assess the potentialities of this compound in order to obtain anti-Stokes laser-induced cooling. Bulk thermal measurements performed on the powder sample by using a thermal infrared camera show a very inhomogeneous heat distribution at the sample surface due to the random distribution of the pumping energy inside the sample, as well as to the random propagation of the emitted thermal field. The analysis of both spectroscopic and thermal measurements shows that after a transient heating induced by the background absorption, cooling of discrete regions can be attained by means of anti-Stokes processes.

From the point of view of potential applications, it is worth noticing that although the relative cooling in these regions is small, the cooling processes involved in the rare earth-doped samples may be enough to avoid the burning of the sample at high excitation fluencies, as has been experimentally proved by comparing the behavior of doped and undoped samples.

## Figures and Tables

**Table 1:** Lifetimes of the ^4^I_9/2_, ^4^I_11/2_, and ^4^I_13/2_ levels obtained under excitation at 806 nm at room temperature. (*λ*_em_ is the emission wavelength).

*λ*_exc_ = 806 nm				
%mol	*λ*_em_ = 820 nm	*λ*_em_ = 1000 nm		*λ*_em_ = 1530 nm
	*τ* (^4^I_9/2_) (μs)	*τ* (^4^I_11/2_) (ms)	*τ* (^4^I_13/2_) (ms)	
0.5	13	2.5	6.1	
1	13	2.5	5.8	
2	13	2.6	6.1	
3	13	2.6	6.2	
6	12	2.3	4.6	

**Table 2:** Lifetime values of the ^4^S_3/2_ level obtained under excitation at 488 nm and 806 nm.

%mol	*λ*_exc_ = 488 nm ^4^S_3/2_	*λ*_exc_ = 806 nm ^4^S_3/2_		
	*τ* (μs)	*τ*_rise_ (μs)	*τ*_s_ (μs)	*τ_l_* (μs)
0.5	112	19	229	957
1	97	19	184	913
2	76	17	167	880
3	58	13	151	847
6	27	6	91	577

**Table 3:** Lifetime values of the ^4^F_9/2_ level obtained under excitation at 488 nm and 806 nm together with the lifetime values of the ^4^I_11/2_ level.

%mol	*λ*_exc_ = 488 nm ^4^F_9/2_	*λ*_exc_ = 806 nm ^4^F_9/2_			^4^I_11/2_
	*τ* (μs)	*τ*_rise_ (μs)	*τ*_s_ (μs)	*τ_l_* (ms)	*τ* (ms)
0.5	228	105	274	1.5	2.5
1	228	120	277	1.5	2.5
2	224	121	312	1.5	2.6
3	209	95	452	1.6	2.6
6	190	8	382	1.3	2.3
